# Treatment Strategies for Infected Wounds

**DOI:** 10.3390/molecules23092392

**Published:** 2018-09-18

**Authors:** Irina Negut, Valentina Grumezescu, Alexandru Mihai Grumezescu

**Affiliations:** 1 National Institute for Lasers, Plasma and Radiation Physics, Magurele RO-77125, Romania; negut.irina@inflpr.ro (I.N.); valentina_grumezescu@yahoo.com (V.G.); 2 Faculty of Physics, University of Bucharest, Magurele 077125, Romania; 3 Faculty of Applied Chemistry and Materials Science, Politehnica University of Bucharest, Bucharest 011061, Romania; 4 Research Institute of University of Bucharest, ICUB, Bucharest 050107, Romania

**Keywords:** nanoparticles, wound healing, wound infection, antibiotics, antibacterial activity essential oils, Manuka honey

## Abstract

The treatment of skin wounds is a key research domain owing to the important functional and aesthetic role of this tissue. When the skin is impaired, bacteria can soon infiltrate into underlying tissues which can lead to life-threatening infections. Consequently, effective treatments are necessary to deal with such pathological conditions. Recently, wound dressings loaded with antimicrobial agents have emerged as viable options to reduce wound bacterial colonization and infection, in order to improve the healing process. In this paper, we present an overview of the most prominent antibiotic-embedded wound dressings, as well as the limitations of their use. A promising, but still an underrated group of potential antibacterial agents that can be integrated into wound dressings are natural products, especially essential oils. Some of the most commonly used essential oils against multidrug-resistant microorganisms, such as tea tree, St. John’s Wort, lavender and oregano, together with their incorporation into wound dressings are presented. In addition, another natural product that exhibits encouraging antibacterial activity is honey. We highlight recent results of several studies carried out by researchers from different regions of the world on wound dressings impregnated with honey, with a special emphasis on Manuka honey. Finally, we highlight recent advances in using nanoparticles as platforms to increase the effect of pharmaceutical formulations aimed at wound healing. Silver, gold, and zinc nanoparticles alone or functionalized with diverse antimicrobial compounds have been integrated into wound dressings and demonstrated therapeutic effects on wounds.

## 1. Introduction

A wound can represent a simple or a severe disorder to an organ (such as the skin) or a tissue and can spread to other tissues and anatomical structures (e.g., subcutaneous tissue, muscles, tendons, nerves, vessels, and even to the bone) [1]. Among all human body (HB) organs, the skin is without doubt the most exposed to impairment and injury, scratches, and burns. By damaging the epithelium and connective structures, the HB’s capability to provide protection from the outer environment is weakened. It is therefore imperative to refabricate a functional epidermis or even other layers of skin. This happens by a cascade of intersecting phases, known as wound healing or wound repair. The repair is reached by the HB’s capacity to substitute lost skin structure with a viable one, and by the formation of a scar.

An improper repair process can cause severe damage, like the loss of skin, initiation of an infection, with consequent harms to the subjacent tissues and even systemic ones [2]. The most common and inevitable impediment to wound healing is the installation of an infection, mostly in the case of chronic wounds. Although bacteria are a common part of the intact skin microbiota and wounds, a critical threshold of existing bacteria and the formation of a biofilm may impede wound healing [3]. Due to these facts, regardless of recent progress in the management of wounds, bacterial and fungal infections are still considered as one of the most collective and painful states which lead to significant mortality and morbidity [4]. *S. aureus*, methicillin-resistant *S. aureus* (MRSA) and *Pseudomonas aeruginosa* are the prevailing microbial strains that occur in patients with infected wounds [5].

Due to the distinctive biological, non-sterile wound environment and the extremely intricate system of wound healing, effective and targeted cures are still needed. Hence, research is currently motivated to find more efficient therapeutics for both chronic and acute wounds infections [6]. In the case of chronic wound occurrence, where individuals frequently endure extensive treatments and regular dressing changes, a fully dissolvable, non-replaceable or non-adherent wound dressing, that distributes treatments to the wound site in a precise manner can improve therapeutic outcomes and the wound response to drugs [7].

Wound dressings are conventionally only applied to shield the wound from external contamination, but they could also be functionalized with different therapeutic complexes to be delivered to wound sites [8]. In contrast to traditional dressings (like bandages made from cotton and wool) that have no active function in the healing process, advanced wound dressings can be designed to take part in it, by means of incorporated active ingredients [9]. The integrated complexes must show a dynamic role in the wound healing process either as helping the removal of necrotic tissues, preventing/treating installed infections, or both. In this respect, dressings can be functionalized with many classes of antibiotics (such as quinolones [10], tetracyclines [11], aminoglycosides [12], cephalosporins [13], etc.) or other substances that exhibit antibacterial properties (e.g., essential oils) [14,15]. Antibiotic-embedded wound dressings are valuable in the management of local infections where high concentrations of antibiotics are needed locally [16].

However, in some cases, high amounts of antibiotics can lead to systemic toxicity [17]. The development of new antibiotics has decreased over the last years, with a small number of companies remaining active in these domains. In addition, antibiotic-resistant microorganisms have considerably increased, due, among others, to the overuse and misuse of antibiotics [18]. This antibiotic crisis is still in progress and affects antibiotic treatments used for both systemic and topical infections. For example, the treatment of chronic wounds (in case of diabetic foot, venous ulcers, and pressure ulcers) habitually involves long-term therapy. Given current problems posed by these infections, the antimicrobial potential of unconventional, non-antibiotic treatments has a renewed interest. In particular, the role of natural agents, especially essential oils [19,20] and honey, in wound-healing has attracted the scientific community as well as some companies for producing wound dressings [21].

Moreover, nanotechnology can be a genuine support in circumventing high-systemic doses. Nanotechnology represents a developing field that manipulates materials in the nanometer size range or molecular/atomic scale, and those materials can have many applications in regenerative medicine and preventing various diseases [22]. When reducing a material’s size to nanoscale, its surface- to- area/-volume ratios will intensely increase, resulting into advantageous physio-chemical reactivity for countless medical applications. Some examples of medical uses include the developing of novel drugs and more operative drug delivery platforms, in vivo imaging, nanobiosensors, wound healing, etc. [23]. In nanomedicine, metal nanoparticles (NPs) such as silver (Ag) [24], gold (Au) [25] and zinc (Zn) [26] show impressive properties like low in vivo toxicity, and bacteriostatic/bactericidal activity [27]. These convenient properties recommend their integration into wound dressings.

This paper aims at reviewing noteworthy in vitro and in vivo results in the area of wound dressings embedded with active ingredients like antibiotics, essential oils (EO) and honey that prevent and/or treat infected wounds. In the same time, we have collected and included recent data that underlines the matchless potential of NPs-based wound dressings that optimize the therapeutic outcomes of wound dressings.

## 2. Wound Healing Process and Skin Wound Microbiology

The skin is the largest organ of the HB and represents a defense shield against mechanical, chemical and biological agents, and ultraviolet radiation. Also, it presents excessive water loss, provides hydration and temperature regulation [28]. There are three multi-histological layers of the skin, which are the epidermis, the dermis and the subcutaneous tissue, as well as skin adjuncts (like hairs and glands), which grow from the profound dermis to superficial epidermal layers [29].

**Figure 1:**
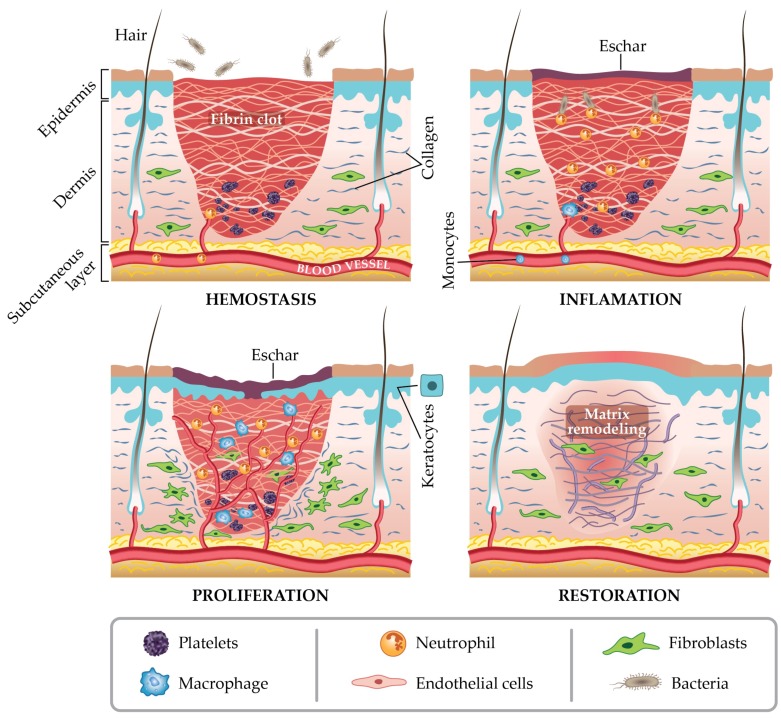
The four stages of wound repair.

The wound healing, in any tissue, is a normal biological process and it involves four complex steps: homeostasis/coagulation; inflammation, migration and proliferation; re-epithelialization and restoration [30] (Figure 1).

Each phase of the wound healing process is influenced by a series of essential mediators, like platelets and cytokines, inflammatory cells, cellular and extracellular matrix, proteinases, growth factors and inhibitors [31]. Usually, the hemostatic and inflammatory stages take place immediately after damage, but the inflammatory stage may last for up to 6 days [2]. The proliferation stage is considered as the beginning of angiogenesis and the development of the extracellular matrix [32]. A prolonged time of the inflammatory and/or proliferative phase will result in a hindered healing, encouraging excessive scar tissue establishment [33]. The remodeling stage typically initiates 3 weeks after damage and can take up to 2 years to fully settle [34]. Many details of the complex process of wound healing can be found in a recent report by Martin and Nunan [35]. A comprehensive consideration of this sequence of stages, cells involved in each stage, time table, and molecular signaling, can consent for the maximum optimization of the healing treatment.

Wounds can be classified as acute and chronic. Acute wounds represent the injured skin (e.g., resulted from burns and chemical injuries) that heals through the regular phases of wound repair; in contrast, chronic wounds need a longer healing time. This longer healing time can be usually attributed to many factors, including amplified levels of inflammatory mediators, wound infection, hypoxia and poor nutrition [36]. In the same time it can be dependent on the patient’s age [37] or underlying comorbidities (such as diabetes, wound dryness) [38].

The skin microbiota diversity and the cutaneous microenvironment (dry, moist, and sebaceous) can influence the wound repair process and the occurrence of skin infections [39]. There exist four prevailing bacterial phyla on the skin: Actinobacteria, Proteobacteria, Firmicutes, and Bacteroidetes that arrange into biofilms and actively take part in the inhibition of skin infections [40].

**Figure 2:**
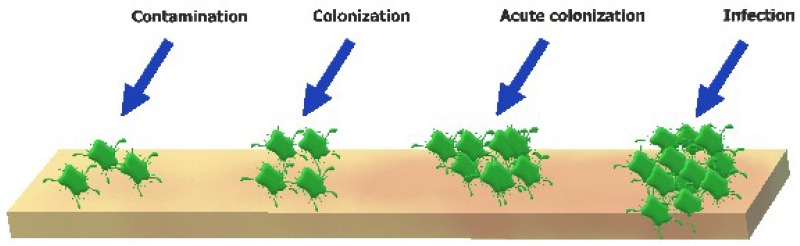
Illustration of the continuum from contamination to wound infection. *Contamination* is represented by the existence of non-replicating bacteria and is a common circumstance in chronic wounds; wound contamination does not promote impaired healing. *Colonization* is the occurrence of replicating microorganisms without triggering host responses. *Acute colonization* is characterized by the manifestation of replicating bacteria resulting in moderate local reaction; this increase of bioburden can delay wound healing. *Infection* occurs when microorganisms are multiplying and have entered the tissue, producing a systemic host response.

As soon as the skin is impaired, typical microorganisms of the normal skin flora and exogenous bacteria and fungi can soon gain access to underlying tissues, which offers a humid, warm and nutrient rich environment [41] for their development. However, when the healing is delayed, the normal microbiota of the wound changes and more aggressive microbial types are hosted [42]. Therefore, an open wound can be a favorable place for microbial proliferation and colonization. In the initial phases of chronic wound construction, Gram-positive entities, mostly *S. aureus*, appear the most. In advanced phases, Gram-negative species (e.g., *Escherichia coli* and *Pseudomonas* sp.) are mostly present and are likely to enter the deeper skin layers, significantly affecting tissues. Additionally, cocci types are present in ~50% of chronic wounds [5]. An infection at a wound site starts with contamination, and continues with (acute) colonization and wound infection (as represented in Figure 2).

Neither contamination nor colonization of a wound initiate an immune response. Besides the multiplication of microbial cells, acute colonization is typically linked to amplified local pain and the start of an inflammatory reaction [43]. By the invasion of healthy tissues by microorganisms, a cascade of local and systemic host reactions is created, for instance purulent expulsion, spreading erythema, or symptomatic cellulitis [3].

As mentioned, the unlimited development of microorganisms can delay wound closing due to an aggravated and extended inflammatory stage. The problematic pathogens are summarized in Table 1.

Infection in chronic wounds is frequently polymicrobial, boosting synergistic effects (both aerobic and anaerobic microorganisms sustain the persistence and multiplication of each other). This collective effect can be usually supported by oxygen consumption; aerobic bacteria can encourage tissue hypoxia, building advantageous conditions for anaerobic multiplication. Once anaerobic species are established, they can obstruct phagocytosis of other microorganisms by producing short chain fatty acids. Furthermore, the nutrient flux from one bacteria may sustain the evolution and proliferation of another [3]. In many types of chronic wounds, *S. aureus* and *P. aeruginosa* are usually encountered growing simultaneously in co-cultures [42]. Numerous pathogens can stick together, forming biofilms, which are microbial masses surrounded by a polymeric environment, consequently evading the killing activity of antibiotics and host effectors. Biofilms can be considered as a physical obstruction to wound healing where the usual process of the inflammatory phase may be extended. It has been reported that bacterial by-products in the form of fatty acids can impede chemotaxis of neutrophils and phagocytosis of *E. coli* and *S. aureus* bacterial cells [55,56]. MRSA infections make the body susceptible to other forms of bacterial and fungal infections and, in time, lead to the creation of multi-species wound infections which are problematic to eliminate [57]. For example, MRSA represents 40% of wound isolates and infects 14–17% of patients experiencing burn wounds [58]. Moreover, it was found that in many cases, *Candida* sp. co-infects burn wounds [59].

In an immunocompromised individual, the bacteria can enter the deeper tissues. With the purpose of accelerating wound healing, topical antimicrobials can promote the treatment of severely infected wounds. Both in vitro tests and data collected from patients indicate that antimicrobial wound dressings can be helpful in wounds that may be affected by biofilms [60,61].

Although antibiotics have significantly improved the health of human beings by treating infections, many of the above mentioned infectious strains still cause a substantial problem worldwide (both in hospital and community settings), by means of antimicrobial resistance [4]. As antimicrobial resistance is on the rise, new antimicrobials in the form of natural products, modified antibiotics, nanoparticles, etc., can add a positive reaction to the trial of antimicrobial-resistant pathogens.

## 3. Ideal Properties of Wound Dressings

To address the health issue associated to wound infections, different forms of wound dressings have been developed in order to protect the wound from contamination and also to accelerate wound healing. Lately, traditional wound dressings (such as bandages of cotton and wool) which passively offer wound safety, have been substituted by innovative dressings that are proficient in providing a favorable environment and distributing active ingredients to the wound to facilitate wound healing. In this respect, a plethora of materials and combinations, both synthetic or natural, have been utilized, with numerous formulations (e.g., sponges [10], hydrogels [62], films [63,64,65], hydrocolloids [66], hydrofiber mats [67,68], etc.) and properties that make them appropriate for the management of a specific wound. Based on their nature of action, wound dressings are classified into three main groups: inert/passive, bioactive, and interactive (Table 2).

**Figure 3:**
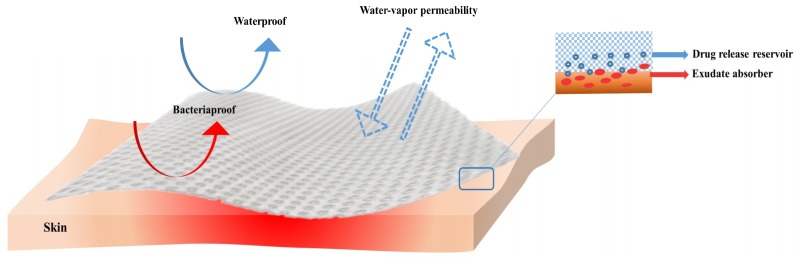
Properties of an ideal wound dressing.

When in contact with the wound, the dressings must provide a moist environment while absorbing wound fluids [69,70,71], and maintain appropriate tissue temperature to improve the blood flow to the wound [72]. The dressings must be biocompatible [73], semi-permeable to water and oxygen [74], promoting tissue renewal processes, hypoallergenic while not provoking immune responses [72] (Figure 3). Furthermore, the dressing must not produce traumas when removed and must also be cost effective. One may conclude that due to diverse features of each wound, including healing stages, there is no dressing that can be appropriate in all circumstances. Nevertheless, many research teams fabricated and improved different wound dressing materials that fit the most of needs for particular wound phases [75].

Recent progresses have been dedicated to adding antimicrobial/antibacterial agents into the traditional wound dressing itself. Wound dressings that contain antibacterial agents are not proposed for the removal of a spreading infection (which generally involves a systemic antibiotic treatment), or for treating uninfected wound beds. Still, they are mostly suitable in a locally infected wound [85].

## 4. Antibacterial Agents in Wound Dressings

### 4.1. Antibiotics

Many studies revealed that various bacteriostatic or bactericidal antibiotics can assist wound closing, still their positive influence on wound healing is regularly unnoticed. Even if countless antibiotics are known to be effective against infection-producing microorganisms, merely quinolones, tetracyclines, aminoglycosides and cephalosporins have been applied to produce antimicrobial wound dressings. Table 3 summarizes the antibiotics-containing wound dressings and their spectrum of action.

These classes of antibiotics can obstruct some functions and/or the metabolic path of the bacteria through one of the following four pathways: inhibition of bacterial cell wall synthesis, blockage of key metabolic pathways, interference on protein synthesis, and inhibition of nucleic acids synthesis [93,94]. Even though many antibiotics are useful in the treatment of infected wounds, their repeated and/or improper usage can initiate bacterial resistance [95]. It was found that about 70% of bacteria that cause wound infections are resistant to minimum one of the most commonly used antibiotics [96]. According to a study performed on 470 samples of wound secretions with bacteriological identification, *S. aureus* and *P. aeruginosa* strains were both significantly resilient to antibiotics application [97]. In addition, infectious strains are starting to acquire resistance to almost all classes of antibiotic, leading to an imperative need for finding new healing substitutes, such as EO [98] and NPs [95].

### 4.2. Natural Antimicrobials for Wound Infections

As antibiotics are gradually becoming tolerated by infection-producing strains, people are now appealing to the vast repertoire of bioresources. These are principally herbs but may include animal and mineral ingredients. Many natural agents that have activities on wounds complicated by polymicrobial infections have been reported in the literature [99]. Their bactericidal outcomes (at the biofilm level) target both initial and advanced phases of wound infection [100].

This section contains recent published literature that refers to the application of EO and other natural alternatives, like honey, as regenerative and antibacterial agents that accelerate wound healing processes. Many in vitro and in vivo studies validate the antimicrobial and regenerative properties of essential and/or edible oils and honey.

#### 4.2.1. Essential Oils

EO, also termed as “volatile natural mixtures”, are plant secondary metabolites that possess antioxidant, anti-inflammatory, anti-allergic, antiviral, antimicrobial and regenerative properties [101]. The EO are mostly synthesised from vegetable parts of plants (such as leaves, seeds, barks, twigs and roots).

Various studies pointed towards the fact that the antimicrobial action of EO integrated in wound dressings can be ascribed to their different constituents (like cinnamaldehyde, geraniol, thymol, menthol and carvacrol, etc.) [21,102,103]. The amount and the presence of these constituents into EO is highly dependent on the extraction procedure (e.g., hydrodistillation, microwave assisted extraction, steam distillation, microwave-generated hydrodistillation, microwave steam diffusion and ultrasound assisted extraction) [19], and the sample source [20]. Diverse published papers on EO that designate the antimicrobial activity of EO to phenolic compounds (specifically to thymol and carvacrol) explain in detail the mechanism through which these compounds act on bacteria. For example, Kavoosi et al. [104], stated that EOs attack lipids and phospholipids existing in cell membranes and in the bacteria cell wall, causing cytoplasm outflow, pH decrease, and impairment of cellular processes (such as ATP biosynthesis, DNA transcription and protein synthesis). Other group described that EOs disrupt the purpose of the cytoplasmic membrane, by interfering with the dynamic transport of nutrients through the cell membrane, and coagulation of bacteria cell matters [105].

The great benefit is that EOs have no/little effect on the development of antimicrobial resistance compared to antibiotics [106]. Moreover, there is a plethora of in vitro tests that point towards using EO as innovative treatments for multidrug resilient microorganisms [107,108]. For example, EOs of thyme, peppermint, lavender, cinnamon, tea tree, rosemary, eucalyptus, lemongrass, etc., have been found to have antimicrobial properties [109]. In addition to antibiotics and antiseptics, EOs have been integrated in wound dressings to function as antibacterial agents [110,111]. However, despite their helpfulness in treating wound infections, one may require a repetitive application and/or high concentrations that may result in adverse effects on the patient.

*Melaleuca alternifolia*, commonly known as tea tree, is a well reputed plant in folk and traditional remedies and remains of particular interest in modern medicine due to its prolonged historic status as a healing agent. Now, useful properties of the tea tree EO and its components have been alternatively integrated into various products, such as dermatological creams and ointments. Tea tree EO, extracted from the leaves and terminal branches of the *Melaleuca alternifolia*, is composed from a mixture of ~100 different components, mostly monoterpenes and sesquiterpenes [112], from which terpinen-4-ol and 1,8-cineole are the most active (analgesic, antiviral, antibacterial, antifungal, antiprotozoal, anti-inflammatory).

There are encouraging data for the treatment of wound infections with tea tree EO integrated into various functional dressings. For example, an electrospun polycaprolactone (PCL) nonwoven mat covered with a layer of chitosan and containing tea tree EO were produced and investigated in vitro [113]; tests proved that the mat effectively inhibited the growth of *S. aureus*. In vivo tests were performed by skin subtraction having dimensions of (1.2 × 1.2) cm^2^ on the back of the C57BL6/J mice in non-infected and infected animal models. After 7 days post-treatment, bacterial levels were found to be lower for the nonwoven mat with 10 μL concentration of tea tree EO-treated groups than the control: 81.6 ± 18.1% and 93.7 ± 9.57% decrease of bacterial contents, as compared to the control group [113]. Other studies synthesized films of chitosan loaded with different contents of tea tree EO droplets. The obtained films were tested in vitro against common wound pathogenic bacteria and fungi, *S. aureus*, *E. coli* and *C. albicans*, respectively. The present study reported that films exhibited antimicrobial activity against all strains and this activity is enhancing with the content of tea tree EO in films. Furthermore, films were non-toxic to L929 fibroblasts and sustained their growth [114].

Tea tree EO was evaluated in a randomized controlled trial on chronic human wounds complicated by MRSA strains. In an uncontrolled, open-label, pilot study for testing the effectiveness of tea tree EO, Edmondson et al. [115] enrolled 19 patients to suffering from MRSA infected wounds. From 19 patients, 11 were treated with a water-miscible tea tree oil (3.3%) solution which was applied at each dressing change. Even though 8 out of 11 wounds were reduced in size after treatment, the infection was not fully eradicated. In a recent study, Lee et al. [116] used 10% of topical tea tree preparation for chronic wounds colonized with MRSA in a nursing home residents. Infections occurred in 14 out of 16 patients treated with tea tree EO, were entirely eradicated after 4 weeks of treatment. Additionally, regarding the wound healing process, 16 colonized wounds in the tea tree oil group closed in 28 days, as compared to the control group. Moreover, tea tree EO is used to produce a commercially available hydrogel dressing, namely Burnaid ^®^ (Mundipharma Pty Limited, Sydney, Australia), used for the treatment of burns [117].

*Hypericum perforatum* (St John’s Wort) has been used since ancient times for its beneficial effects on combating depression, anxiety, inflammation, microbes, and pain [118]. In the last decades, the plant has been studied for its constituents such as hypericin, hyperforin and flavonoids. The antibacterial activity of *Hypericum perforatum* it attributed to hyperforin. Moreover, it was found that the same hyperforin has a major role on the reepithelization of the skin [118]. As a result, the EO of *Hypericum perforatum* has become one of the most operative and extensively spread herb for wound therapy [118]. Different concentrations (0.25–1.5% *v*/*v*) of *Hypericum perforatum* oil were incorporated into chitosan films for wound dressing applications. In vitro test performed on *E. coli* and *S. aureus* strains showed that chitosan—*Hypericum perforatum* oil based films had antimicrobial effect on both microorganisms [119].

*Lavandula angustifolia* (commonly known as lavender) has been used in traditional medicine worldwide. It has been proved that the EO of lavender efficiently inhibits growth of infection-producing microorganisms [120]. The antimicrobial activity of lavender EO (LO) was attributed to its major components, linalool and linalyl, but the chemical composition and antibacterial action of lavender EO is mainly dependent on the lavender samples source [121]. In this respect, an interesting study has been presented by Imane et al. [122] who reported on the chemical composition and antibacterial effect of lavender EO. Their EO confirmed the presence of high amounts of linalool, linalyl acetate, 1,8-cineole, and camphor. Moreover, in vitro tests demonstrated a strong antibacterial activity and inhibition against *E. coli* ATCC 25922, *S. aureus* ATCC 25923 and *P. aeruginosa* ATCC 27853, with a minimal inhibitory concentration of 3.33, 1.33 and 42.67 μL/mL [122]. Nevertheless, lavender EO was proposed to be used for the treatment of surface infection in the form of a prophylactic or topical application [121].

Besides the demonstrated antibacterial and antifungal activity, lavender EO also plays an important role in improving wound healing phases. It is noteworthy to mention a study by Mori et al. on topical application of LO on a wounded rat model in which LO encouraged wound healing in the primary phase by stimulating collagen production and differentiation of fibroblasts and by quickening the establishment of granulation tissue [123].

The *Origanum* genus (commonly known as oregano) has ~39 species, from which *Origanum vulgare* L. is the most considered in many scientific studies [124]. Oregano EO has been reported to have several biological activities, like antioxidant, antimicrobial, and antimutagenic, which are correlated with the presence of thymol and carvacrol in its composition [124]. Many in vitro and in vivo studies have shown that EO can be used in the treatment of *S. aureus*, *MRSA* and *E. coli* bacterial infections [125]. In a work by Liakos et al., cellulose acetate electrospun fibers have efficiently incorporated oregano EO for applications as improved antimicrobial wound dressings. Moreover, while analyzing the in vitro development of bacteria and biofilms, the authors observed the inhibitory effect on all tested strains at all studied EO concentrations (1% and 5%), but this effect is significant when the amount of 5% oregano EO was used for the design of cellulose acetate fibers. Tests performed on fibers formed with oregano EO revealed a very good antibacterial activity against all tested microbial strains with the potency decreasing as follows: *C. albicans* > *E. coli* > *S. aureus* [126].

#### 4.2.2. Honey

Honey has been used for decades as a natural healing agent for many human diseases for instance cardiovascular and gastrointestinal tract ailments, and infections of upper respiratory tract, as well as in infected wounds [127]. The therapeutic properties of honey such as the capability to provide a topical nutrition to the wound, inflammation reduction, granulation and angiogenesis stimulation, wound epithelialization, recommend it to be integrated into wound dressings [21]. The research carried out during last decades demonstrated that honey’s bacteriostatic and bactericidal activity can be ascribed to several factors:

- It’s acidic pH (regularly in the range of 3.4–6.1). It has been found that the acidic character of honey may encourage macrophages to eradicate bacteria and inhibit microbial biofilm establishment [128].
- The osmotic pressure applied by sugars found in its chemical composition. The high osmolality obstructs microbial development [129].
- The presence of antibacterial components such as hydrogen peroxide, antioxidants, lysozyme, phenolic acids, flavonoids, methylglyoxal and bee peptides (such as defensin-1) [130,131]. The production of hydrogen peroxide is a crucial component for the inhibition of bacterial development. In particular, hydrogen peroxide is gradually released/formed when the wound exudate interrelates with glucose oxidation, triggering the oxidative damage to pathogens’ macromolecules; hydrogen peroxide can react with the bacterial cell wall, as well as with intracellular lipids, proteins and nucleic acids [132].

Taking into account the abovementioned, studies on different honey types revealed their high efficiency against the most prevalent microorganisms that are involved in wound infections. The investigations published by Kuś and coworkers [133] have shown that from 14 honey varieties from Poland analyzed for their antimicrobial activity, cornflower, buckwheat and thyme honeys were the most active against the growth of *S. aureus* PCM 2051 strain at concentrations of 3.12 or 6.25% (*v*/*v*). In the same study, adequate action was observed for linden tree, heather, savory and coriander honeys. Likewise, growth inhibition of a broad range of MRSA microorganisms has been reported by using of Ulmo tree [134], melaleuca [135] and longan flower [136] honeys, from many geographical regions.

However, in the presence of catalase- an enzyme that reduces the hydrogen peroxide- honey displays a diminished antimicrobial action. Moreover, the composition of honey is dependent on the floral source, bee species and geographical setting [137]. To surpass this limitation, and problems generated by “traditional honeys” (such as the presence of spores that results in deactivation of glucose oxidase) only honeys with certified activities are recommended to be applied in medical domains. Therein, an assortment of medical honeys have entered the market (for example chestnut, manuka, thyme, revamil) [138] which exhibit, by comparison with “traditional honeys” good predictability and quality. At present, some companies are focused on producing dressings containing honey (mostly Manuka honey): Actilite^®^, Algivon^®^, MediHoney^®^, and Activon Tulle^®^ [21].

Manuka honey, which is obtained from the Manuka tree and comprises a non-peroxide, non-degradable by the action of catalase component, can sustain its antibacterial activity in biological fluids [21]. The antibacterial properties exerted by Manuka honey are not only attributed to the hydrogen peroxide but also to the high amounts of the antibacterial compound, methylglyoxal, present in its chemical composition [137]. It was found that Manuka honey impedes the growth of MRSA and *S. pyogenes*, along with tested gram-negative strains (such as *E. coli*, and *P. aeruginosa*) [139] and eludes biofilm establishment on the wound site [140]. Considering the beneficial multifaceted properties of this specific type of honey, in terms of the anti-inflammatory activity, wound repair efficacy and antibacterial properties, many research groups fabricated Manuka honey dressings. Minden-Birkenmaier et al. focused their study on fabrication and characterization of Manuka honey-containing poly(e-caprolactone) (PCL) nanofiber scaffolds to be applied as wound dressings and precursors to tissue-engineered skin. The obtained results confirmed that honey positively influenced in vitro fibroblasts infiltration into the scaffold, while inhibiting the growth of *E. coli* strain [141]. Moreover, Yang and coworkers, incorporated Manuka honey as a functional antibacterial agent in an electrospun membrane produced with silk fibroin. The obtained fibrous matrices exhibited antibacterial activity against MRSA and methicillin-susceptible *S. aureus*, *E. coli* and *P. aeruginosa* [142]. In a study by Tavakoli et al., a highly concentrated honey-Poly(vinyl alcohol) hybrid hydrogel was produced not only to promote antibacterial activity, but also to prove its biocompatibility. It was observed that the honey/Poly (vinyl alcohol) hybrid effectively encouraged the wound healing process by establishing a thin layer of hydrophilic gel that reduces the risk of contamination. Same dressing proved to be effective against *S. aureus* and *E. coli* pathogens [143].

As regarding the clinical benefits of honey, a recent review outlines observation reports and randomized controlled trials, as well as an update of the recently published literature [144]. However, literature reports varied honey’s outcomes depending on the wound type and also it has been shown that honey may even have harmful effects. These incongruous results point toward that more information is needed and large randomized clinical trials are essential to demonstrate the clinical benefit of honey in reducing the prevalence of wound infections.

### 4.3. Nanoparticles

Treating wound infections produced by multidrug-resistant bacteria is a major task owing to the incapability of conventional antibiotics to treat such infections [145]. NPs are considered as promising replacements to habitually applied antibiotics, as they alone demonstrated bactericidal activity against a big number of pathogens, are capable of diminishing side effects of medications and do not produce microbial resistance [27].

When a NP encounters a bacterial cell wall, it can accomplish its bactericidal effect by discharging toxic metal ions or by generating Reactive Oxygen Species (ROS) [146]. When in intimate contact with bacterial cells walls, negatively-charged groups originated at bacteria surfaces attract positively-charged NPs. At that time, van der Waals forces, receptor-ligand, and hydrophobic interactions are established and the cell wall absorptivity is changed by the establishment of “holes” in bacteria’s surfaces [147,148]. In addition, NPs can also penetrate the cell wall, affect metabolic paths, disrupt mitochondria and can also affect proton efflux pumps subsequent with a pH modification and a membrane’s surface charge disorder [146,149]. Bacteria species sensitivity is not only linked to the structure of their cell walls. Extra factors can affect the weakness or tolerance of bacteria to NPs treatment. The mechanisms of NP toxicity are very complicated but are usually determined by the composition, surface modification of NP, intrinsic properties, and bacterial species. For instance, *E. coli* is very vulnerable to CuO and ZnO NPs, while *S. aureus* and *Bacillus subtilis* are less susceptible [150]. Ag NPs antibacterial effect against *E. coli* and *S. aureus* bacterial strains is higher than that of Cu NPs against same bacteria [151,152]. The toxicity of Cu NPs is governed by a combination of several factors (e.g., high temperature, high aeration, low pH, NPs and bacteria concentration) that can increase the toxicity [153]. Amongst CuO and ZnO NPs used against *E. coli*, *B. subtilis*, and *S. aureus*, CuO NPs present the highest toxicity [96,146].

Many of the aforementioned NPs alone or functionalized with diverse antimicrobial compounds (such as antibiotics and natural products) have been used for applications in wound healing. Ag NPs have received extensive consideration by the scientific community owing to their inhibitory action towards ~650 microbe species and against antibiotic resistant bacteria [24]. Ag NPs can inhibit the bacterial reproduction by denaturing bacterial DNA which leads to bacterial cell alteration and finally to cell death [154]. The toxicity of Ag and Ag nanoforms can eradicate microorganisms; likewise, it can have the same effect on healthy human cells. It has been found that Ag has a concentration-dependent cytotoxic effect on human dermal fibroblast cells [155]. With the expansion of nanotechnology, specialists were able to establish a therapeutic window that boosts antimicrobial properties of Ag, and decreases it’s minimum inhibitory concentration as well as reducing toxicity to normal human cells [156]. Therefore, many wound dressings containing Ag (such as Acticoat, Bactigrass, Tegaderm, Fucidin, PolyMem Silver) have been accepted for introduction in the market by the U.S. Food and Drug Administration [157]. Among metallic NPs, Ag NPs are extensively applied in formulating ointments for burns and wound dressings used in pressure ulcers [158,159]. In a latest study using human keratinocytes and dermal fibroblasts, Ag NPs action considerably diminished levels of inflammatory cytokines and encouraged healing [160].

A large number of studies are now focused on formulations of wound dressings made from chitosan hydrogels that incorporate and release nano-Ag and Ag NPs [161,162,163,164,165]. For example, spherical Ag NPs of (10–30) nm were embedded into surfaces of nano-fibers for wound healing applications. This nanostructure demonstrated substantial antibacterial activities with over 99% reductions in *E. coli*, *S. aureus* and *P. aeruginosa* and endorsed the progress of epidermal cells with no cytotoxicity [166]. Our group used Ag NPs to coat polyester-nylon wound dressings for reducing the risk of exogenous wound related infections caused by *P. aeruginosa* and *S. aureus* bacterial strains. The in vitro and in vivo studies proved that tested NPs applied as coating allowed the normal development of cultured fibroblast cells and showed toxicity within a mouse model. The modified and viable cells count analyses proved that the modified wound dressing had an enhanced inhibitory activity against bacterial colonization, and biofilm growth, especially against *P. aeruginosa* [167].

*Zinc* is active against some antibiotic-resistant microorganisms owing to its intricate antibacterial mechanism [168,169]. *Zinc oxide (ZnO)* represents the core form studied for its antibacterial effect. ZnO NPs are capable of yielding ROS that impede *E. coli* and *S. aureus* biofilm development [170,171,172]. Moreover, it was reported that ZnO NPs, at proper concentrations, have antibacterial activity without affecting normal cells [26,173]. When ZnO was embedded into a chitosan-dextran hydrogel, it proved to have proper antimicrobial properties and be an appropriate component in wound dressings [174]. Knorasani et al. produced nanocomposite dressings by incorporating ZnO NPs into heparinized PVA/chitosan/hydrogels. The results showed this composite dressings’ aantibacterial effect against *E. coli* and *S. aureus* was enhanced with more than 70% by adding ZnO NPs into the hydrogels, as compared to the sample without ZnO NPs. Furthermore, in vitro tests performed on mouse fibroblast cells revealed that the nanocomposites are fully biocompatible [175].

Our group created a novel nano-modified coating for wound dressings, constructed on functionalized ZnO nanostructures and orange oil. The antibacterial efficiency of obtained nanocomposite was tested against *E. coli* and *S. aureus*. Results revealed the effectiveness of obtained nanostructures against both planktonic and adherent bacteria, but the effect was more intense against the *S. aureus* strain [176]. In another study [177], castor oil was used as a matrix material for the development of nanocomposite films filled with different amounts of chitosan-modified ZnO nanoparticles. The microbicidal properties of developed films were evaluated against *E. coli*, *S. aureus* and *Micrococcus luteus*. The antibacterial action towards the last two strains was stronger than that towards *E. coli*. Furthermore, cell viability records shown that composites with chitosan—ZnO loading ≤ 5.0 wt % do not present toxic effects against the tested human dermal fibroblasts. The in vivo healing study demonstrated that wounds treated with castor oil/chitosan-ZnO healed much earlier than castor oil- and gauze-covered wounds, and exhibited a higher content and better aligned collagen arrangement.

*Au NPs* are well known to be biocompatible and are extensively used in biomedical domains [25,178,179,180]. The nanoforms of Au alone do not possess antimicrobial activity and must be joined with other chemical species to be effectively applied in antimicrobial applications [181]. For example, AuNPs can be linked with gelatin, chitosan and/or with collagen to be used in wound healing [182,183,184]. In a study [185], Au NPs were combined with cryopreserved human fibroblasts and topically applied to burn wounds; treated wounds exhibited a greater healing rate, reduced inflammatory stage and amplified collagen installation. In other research, Au NPs were loaded into *N*,*N*,*N*-trimethyl chitosan/alginate complex; this nanocomposite presented a good biocompatibility and a high wound dressing prospective [186].

Although there are many methods for producing clinically safe NPs, their extrinsic properties can cause damage to the human body. In common with various drug treatments, the overexposure to NPs could lead to undesirable side effects. For example, the dispersal and accumulation of NPs into body organs (such as the brain, lungs, kidneys and skin) can trigger toxicity to the host. In order to avoid these risks, in vivo bio-distribution and safe degradation profile studies are crucial in advance to the clinical application of non-biodegradable NPs [27]. Moreover, these studies are vital for verifying NPs (residing from wound dressings) retention into skin layers, undesirable responses of the skin towards NPs (such as inflammation and foreign body reaction) and to track any subsequent escape of NPs into the bloodstream.

## 5. Conclusions

Non-healing wounds due to infection causes still remain a dare to treat, and therefore to the design of advanced materials that could be used as wound dressings. Recent findings allow for the production of wound dressings that have the potential to become ideal candidates for the delivery of beneficial molecules and/or drugs to the wound site. In particular, in this review, we present the incorporation of several antibiotics, essential oils, honey and inorganic NPs, the last ones allowing the creation of composite materials for multi-pharmacological goals.

## Figures and Tables

**Table 1:** Species of microorganisms instituted in acute and chronic wounds.

Species	Shape	Metabolism	Incidence	Ref.
*S. aureus*	Cocci	Facultatively anaerobic	Chronic wounds	[44]
*S. epidermidis*			Acute wounds	[45]
*Streptococcus pyogenes*		Aerobic	Chronic wounds	[46]
*P. aeruginosa*	Bacilli	Aerobic	Chronic wounds	[47]
*Stenotrophomonas maltophilia*				[48,49]
*E. coli*		Facultatively anaerobic		[50]
*Proteus* sp.				[51,52]
*Klebsiella* sp.				[5]
*Propionibacterium acnes*		Aerotolerant anaerobic	Acute wounds	[53]
*Acinetobacter baumannii*	Coccobacilli	Aerobic	Chronic wounds	[48,54]

**Table 2:** Wound dressing types.

Type of Dressing	Formulation	Advantages (A)/Disadvantages (D)	Some Commercially Available Products	Ref.
**Inert/passive**	Gauzes	(A): Are manufactured in forms of bandages, sponges, plasters and stockings. Display a massive porosity, make available thermal isolation, and sustain a humid background at the wound site. Sponges can be applied directly to the surface of suppurating wounds (D): Can stick to wounds, disrupt the wound bed when removed, are suitable mostly for minor wounds; Sponges suffer from lack of mechanical resistance and they are not fitted for third-degree burns management or wounds with desiccated eschar	Multisorb, Urgotul SSD/S.Ag, Curity, Vaseline Gauze, Xeroform	[76,77]
**Bioactive**	Hydrocolloids	(A): Semi-permeable in the form of solid wafers, can enclose hydroactive particles that swell with exudates or form a gel, can be detached from wounds without difficulty by saline or sterilized water, and are usually considered as painless dressings (highly recommended for pediatrics wound care management). (D): Can be applied in wounds with light to heavy exudate (such as eschars, minor burn wounds and traumatic wounds, sloughing, or granulating wounds), can be cytotoxic, can possess a disagreeable odor, sustain an acid pH at the application site and present a low mechanically strength	DouDERM, Granuflex, Comfeel, Tegasorb	[78,79]
	Alginates	(A): Highly absorbent, hemostatic, applicable for exudating wounds, helpful in debridement of sloughing wounds. (D): Limited use on low exudating wounds, causing dryness and scabbing, should be changed daily	Kaltostat, Algisite, Kaltostat, Sorbsan, Tegagen, SeaSorb, PolyMem	[80,81]
	Collagens	(A): They are in the form of pads, gels or particles and encourage the formation and setting of new- formed collagen in wounds, they absorb exudates, offer a humid environment to wounds; They are easy to apply, non-immunogenic, non-pyrogenic, (D): Not recommended to application to wounds with necrosis and third-degree burns; require a secondary dressing	Puracol Plus, Triple Helix Collagen, Cutimed Epiona Sterile, BIOSTEP	[67,82]
	Hydrofibers	(A): Soft nonwoven pad or ribbon dressings that absorb exudates and provide a moist environment in a deep wound together with a reduced risk of skin maceration (D): A certain degree of absorption of fluid is required for pH control, however, the absorption of an excessive amount can cause an undesirable swelling of the wound dressing, leading to distension and possible loss of adhesion;	Aquacel	[67]
**Interactive**	Hydrogels	(A): Rehydrates dry wounds, easy removal/changes, high capacity to accumulate/absorb large volumes of water inside their 3D polymeric network, moist-absorbent wound dressings, permeable to metabolites, non-irritant, and non-reactive with biological tissues (D): May cause over-hydration, weak mechanical properties, consequently necessitating a secondary dressing	Carrasyn, Curagel, Nu-Gel, Purilon, Restore, SAF-gel, XCell	[62,83]
	Semi-permeable films	(A): Semi-permeable, transparent for allowing wound check, highly elastic, and can follow any contour and do not have need of extra patter; waterproof and permeable to oxygen (D): Mostly suitable for superficial wounds with little exudates and for wound epithelialization, used as an additional layer for hydrogels and foams	Opsite, Tegaderm, Biooclusive, Polyskin	[72]
	Semi-permeable foams	(A): Soft, open cell, hydrophobic, usually made from polyurethane sheets; large amounts of exudates. (D): Can cause dryness and scabbing when applied to low exudating wounds and dry scars	Allevyn	[84]

**Table 3:** Antibiotics contained within wound dressings.

Class	Name	Wound Dressing Material	Tested Strains	Ref.
Quinolones	Amoxicillin	Flexible sponges from bacterial cellulose	*E. coli*, *C. albicans* *S. aureus*	[10]
	Ciprofloxacin	Calcium alginate films	*E. coli* *S. aureus* *P. aeruginosa*	[86]
		Films and nanofiber mats of Povidone	*E. coli* *Bacillus subtilis*	[87]
		Electrospun fibers based on thermoresponsive polymer poly(*N*-isopropylacrylamide), poly(l–lactic acid–co-ɛ-caprolactone)	*E. coli* *S. aureus*	[88]
		Hydrogels from 2-hydroxyethyl methacrylate/citraconic anhydride–modified collagen	*S. aureus*	[89]
Tetracyclines	Tetracycline	Cotton fabric coated with chitosan-Poly(vinyl pyrrolidone)–PEG	*E. coli* *S. aureus*	[11]
	Doxycycline	Poly(acrylic acid) nanofiber mats	*S. aureus Streptococcus agalactiae*	[90]
Aminoglycosides	Gentamicin	Thin films made from collagen, chitosan and hyaluronic acid	*E. coli* *S. aureus* *P. aeruginosa*	[91]
		Sodium carboxymethyl cellulose loaded with antibiofilm agents (xylitol and ethylenediaminetetraacetic acid)	*S. aureus* *Bacillus subtilis* *P. aeruginosa* *E. coli*	[12]
	Kanamycin	Nanofibers prepared with a combination of polyethylene oxide and hyaluronic acid	*Listeria monocytogenes* *P. aeruginosa*	[92]
Cephalosporins	Cefuroxime and Cefepime	Biocompatible nanostructured composite based on naturally derived biopolymers (chitin and sodium alginate)	*E. coli* *S. aureus*	[13]

## References

[1] van Koppen C.J., Hartmann R.W. (2015). Advances in the treatment of chronic wounds: A patent review. Expert Opin. Ther. Pat..

[2] Sorg H., Tilkorn D.J., Hager S., Hauser J., Mirastschijski U. (2017). Skin Wound Healing: An Update on the Current Knowledge and Concepts. Eur. Surg. Res..

[3] Bowler P.G., Duerden B.I., Armstrong D.G. (2001). Wound microbiology and associated approaches to wound management. Clin. Microbiol. Rev..

[4] World Health Organization Who Publishes List of Bacteria for Which New Antibiotics Are Urgently Needed. https://tinyurl.com/kmva5da.

[5] Cardona A.F., Wilson S.E. (2015). Skin and soft-tissue infections: A critical review and the role of telavancin in their treatment. Clin. Infect. Dis..

[6] Vyas K., Vasconez H.C. (2014). Wound healing: Biologics, skin substitutes, biomembranes and scaffolds. Healthcare.

[7] Kopecki Z., Cowin A.J. (2017). Fighting chronic wound infection—One model at a time. Wound Pract. Res. J. Aust. Wound Manag. Assoc..

[8] Jones V., Grey J.E., Harding K.G. (2006). Wound dressings. BMJ.

[9] Mirza A. (2018). Nanomedicine and advanced technologies for burns: Preventing infection and facilitating wound healing. Adv. Drug Deliv. Rev..

[10] Ye S., Jiang L., Wu J., Su C., Huang C., Liu X., Shao W. (2018). Flexible amoxicillin-grafted bacterial cellulose sponges for wound dressing: In vitro and in vivo evaluation. ACS Appl. Mater. Interfaces.

[11] Anjum S., Arora A., Alam M.S., Gupta B. (2016). Development of antimicrobial and scar preventive chitosan hydrogel wound dressings. Int. J. Pharm..

[12] Anjum A., Sim C.H., Ng S.F. (2018). Hydrogels Containing Antibiofilm and Antimicrobial Agents Beneficial for Biofilm-Associated Wound Infection: Formulation Characterizations and In vitro Study. AAPS PharmSciTech.

[13] Rădulescu M., Holban A.M., Mogoantă L., Bălşeanu T.A., Mogoșanu G.D., Savu D., Popescu R.C., Fufă O., Grumezescu A.M., Bezirtzoglou E. (2016). Fabrication, Characterization, and Evaluation of Bionanocomposites Based on Natural Polymers and Antibiotics for Wound Healing Applications. Molecules.

[14] Low W.L., Kenward K., Britland S.T., Amin M.C.I.M., Martin C. (2017). Essential oils and metal ions as alternative antimicrobial agents: A focus on tea tree oil and silver. Int. Wound J..

[15] Saporito F., Sandri G., Bonferoni M.C., Rossi S., Boselli C., Icaro Cornaglia A., Mannucci B., Grisoli P., Vigani B., Ferrari F. (2017). Essential oil-loaded lipid nanoparticles for wound healing. Int. J. Nanomed..

[16] Ramasubbu D.A., Smith V., Hayden F., Cronin P. (2017). Systemic antibiotics for treating malignant wounds. Cochrane Database Syst. Rev..

[17] Everts R. (2016). How to Treat Wound Infection. Prevention and Treatment. https://www.acc.co.nz/assets/provider/treating-wound-infections.pdf.

[18] Das P., Horton R. (2016). Antibiotics: Achieving the balance between access and excess. Lancet.

[19] Aumeeruddy-Elalfi Z., Mahomoodally M., Peters M. (2016). Chapter: Extraction techniques and pharmacological potential of essential oils from medicinal and aromatic plants of Mauritius. Essential Oils: Historical Significance, Chemical Composition and Medicinal Uses and Benefits.

[20] Aumeeruddy-Elalfi Z., Gurib-Fakim A., Mahomoodally M. (2016). Chemical composition, antimicrobial and antibiotic potentiating activity of essential oils from 10 tropical medicinal plants from Mauritius. J. Herb. Med..

[21] Scagnelli A.M. (2016). Therapeutic review: Manuka honey. J. Exot. Pet Med..

[22] Zarrintaj P., Moghaddam A.S., Manouchehri S., Atoufi Z., Amiri A., Amirkhani M.A., Nilforoushzadeh M.A., Saeb M.R., Hamblin M.R., Mozafari M. (2017). Can regenerative medicine and nanotechnology combine to heal wounds? The search for the ideal wound dressing. Nanomedicine.

[23] McNamara K., Tofail S.A.M. (2017). Nanoparticles in biomedical applications. Adv. Phys. X.

[24] Zewde B., Ambaye A., Stubbs J., Raghavan D. (2016). A review of stabilized silver nanoparticles—Synthesis, biological properties, characterization, and potential areas of applications. JSM Nanotechnol. Nanomed..

[25] Cabuzu D., Cirja A., Puiu R., Grumezescu A.M. (2015). Biomedical applications of gold nanoparticles. Curr. Top. Med. Chem..

[26] Pelgrift R.Y., Friedman A.J. (2013). Nanotechnology as a therapeutic tool to combat microbial resistance. Adv. Drug Deliv. Rev..

[27] Yang Y., Qin Z., Zeng W., Yang T., Cao Y., Mei C., Kuang Y. (2017). Toxicity assessment of nanoparticles in various systems and organs. Nanotechnol. Rev..

[28] Chua A.W.C., Tan B.K., Foo C.L., Tan K.C., Chong S.J., Khoo Y.C. (2016). Skin tissue engineering advances in severe burns: Review and therapeutic applications. Burns Trauma.

[29] Ho J., Walsh C., Yue D., Dardik A., Cheema U. (2017). Current Advancements and Strategies in Tissue Engineering for Wound Healing: A Comprehensive Review. Adv. Wound Care.

[30] Olivier G., Wael N.H., Gamal B. (2017). Wound healing: Time to look for intelligent, ‘natural’ immunological approaches?. BMC Immunol..

[31] Enoch S., Leaper D.J. (2008). Basic science of wound healing. Surgery.

[32] Xue M., Jackson C.J. (2015). Extracellular Matrix Reorganization during Wound Healing and Its Impact on Abnormal Scarring. Adv. Wound Care.

[33] Rittié L. (2016). Cellular mechanisms of skin repair in humans and other mammals. J. Cell Commun. Signal..

[34] Sanon S., Hart D.A., Tredget E.E., Albanna M.Z., Holmes J.H. (2016). Molecular and cellular biology of wound healing and skin regeneration. Skin Tissue Engineering and Regenerative Medicine.

[35] Martin P., Nunan R. (2015). Cellular and molecular mechanisms of repair in acute and chronic wound healing. Br. J. Dermatol..

[36] Demidova-Rice T.N., Durham J.T., Herman I.M. (2012). Wound healing angio genesis: Innovations and challenges in acute and chronic wound healing. Adv. Wound Care.

[37] Gould L.J., Fulton A.T. (2016). Wound Healing in Older Adults. R. I. Med. J..

[38] Boateng S., Matthews K.H., Stevens H.N.E., Eccleston G.M. (2008). Wound healing dressings and drug delivery systems: A review. J. Pharm. Sci..

[39] van Rensburg J.J., Lin H., Gao X., Toh E., Fortney K.R., Ellinger S., Zwickl B., Janowicz D.M., Katz B.P., Nelson D.E. (2015). The human skin microbiome associates with the outcome of and is influenced by bacterial infection. mBio.

[40] Grice E.A. (2014). The skin microbiome: Potential for novel diagnostic and therapeutic approaches to cutaneous disease. Semin. Cutan. Med. Surg..

[41] Sarheed O., Ahmed A., Shouqair D., Boateng J., Alexandrescu V. (2016). Antimicrobial dressings for improving wound healing. Wound Healing-New Insights into Ancient Challenges.

[42] Serra R., Grande R., Butrico L., Rossi A., Settimio U.F., Caroleo B., Amato B., Gallelli L., de Franciscis S. (2015). Chronic wound infections: The role of *Pseudomonas aeruginosa* and *Staphylococcus aureus*. Expert Rev. Anti-Infect. Ther..

[43] Guo S., Dipietro L.A. (2010). Factors affecting wound healing. J. Dent. Res..

[44] Ortines R.V., Cheng L., Cohen T.S., Gami A., Dillen C.A., Ashbaugh A.G., Miller R.J., Wang Y., Tkaczyk C., Sellman B.R. (2017). Anti-alpha-toxin immunoprohylaxis reduces disease severity against a *Staphylococcus aureus* full-thickness skin wound infection in immunocompetent and diabetic mice. J. Immunol..

[45] Peerayeh S.N., Moghadas A.J., Behmanesh M. (2016). Prevalence of Virulence-Related Determinants in Clinical Isolates of Staphylococcus epidermidis. Jundishapur J. Microbiol..

[46] Regev A., Weinberger M., Fishman M., Samra Z., Pitlik S.D. (1998). Necrotizing fasciitis caused by *Staphylococcus aureus*. Eur. J. Clin. Microbiol. Infect. Dis..

[47] Lu J., Yang M., Zhan M., Xu X., Yue J., Xu T. (2016). Antibiotics for treating infected burn wounds. Cochrane Database Syst. Rev..

[48] Church D., Lloyd T., Peirano G., Pitout J. (2013). Antimicrobial susceptibility and combination testing of invasive *Stenotrophomonas maltophilia* isolates. Scand. J. Infect. Dis..

[49] Brooke J.S. (2012). *Stenotrophomonas maltophilia*: An emerging global opportunistic pathogen. Clin. Microbiol. Rev..

[50] Moet G.J., Jonesab R.N., Biedenbach D.J., Stilwell M.G., Fritsche T.R. (2007). Contemporary causes of skin and soft tissue infections in North America, Latin America, and Europe: Report from the SENTRY Antimicrobial Surveillance Program (1998–2004). Diagn. Microbiol. Infect. Dis..

[51] Kishore J. (2012). Isolation, identification & characterization of *Proteus penneri*—A missed rare pathogen. Indian J. Med. Res..

[52] Mihai M.M., Holban A.M., Giurcăneanu C., Popa L.G., Buzea M., Filipov M., Lazăr V., Chifiriuc M.C., Popa M.I. (2014). Identification and phenotypic characterization of the most frequent bacterial etiologies in chronic skin ulcers. Rom. J. Morphol. Embryol..

[53] Lee M.J., Pottinger P.S., Butler-Wu S., Bumgarner R.E., Russ S.M., Matsen F.A. (2014). Propionibacterium persists in the skin despite standard surgical preparation. J. Bone Jt. Surg. Am..

[54] Howard A., O’Donoghue M., Feeney A., Sleator R.D. (2012). *Acinetobacter baumannii*: An emerging opportunistic pathogen. Virulence.

[55] Rotstein O.D., Vittorini T., Kao J., McBurney M.I., Nasmith P.E., Grinstein S. (1989). A soluble Bacteroides by-product impairs phagocytic killing of *Escherichia coli* by neutrophils. Infect. Immun..

[56] Cutting K.F., White R.J. (2005). Criteria for identifying wound infection revisited. Ostomy Wound Manag..

[57] Felk A., Kretschmar M., Albrecht A., Schaller M., Beinhauer S., Nichterlein T., Sanglard D., Korting H.C., Schäfer W., Hube B. (2002). Candida albicans hyphal formation and the expression of the Efg1-regulated proteinases Sap4 to Sap6 are required for the invasion of parenchymal organs. Infect. Immun..

[58] Katakura T., Yoshida T., Kobayashi M., Herndon D.N., Suzuki F. (2005). Immunological control of methicillin-resistant *Staphylococcus aureus* (MRSA) infection in an immunodeficient murine model of thermal injuries. Clin. Exp. Immunol..

[59] Shirtliff M.E., Peters B.M., Jabra-Rizk M.A. (2009). Cross-kingdom interactions: Candida albicans and bacteria. FEMS Microbiol. Lett..

[60] Beele H., Meuleneire F., Nahuys M., Percival S.L. (2010). A prospective randomised open label study to evaluate the potential of a new silver alginate/carboxymethylcellulose antimicrobial wound dressing to promote wound healing. Int. Wound J..

[61] Percival S.L., Bowler MPhil P., Woods E.J. (2008). Assessing the effect of an antimicrobial wound dressing on biofilms. Wound Repair Regen..

[62] Koehler J., Brand F.P., Goepferich A.M. (2018). Hydrogel wound dressings for bioactive treatment of acute and chronic wounds. Eur. Polym. J..

[63] Dutra J.A.P., Carvalho S.G., Zampirolli A.C.D., Daltoé R.D., Teixeira R.M., Careta F.P., Cotrim M.A.P., Oréfice R.L., Villanova J.C.O. (2017). Papain wound dressings obtained from poly (vinyl alcohol)/calcium alginate blends as new pharmaceutical dosage form: Preparation and preliminary evaluation. Eur. J. Pharm. Biopharm..

[64] Sasikala L., Dhurai B. (2018). Preparation and Analysis of Chitosan-Honey Films for Wound Dressing Application. World Acad. Sci. Eng. Technol. Int. J. Mater. Text. Eng..

[65] Ahmed A., Boateng J. (2018). Calcium alginate-based antimicrobial film dressings for potential healing of infected foot ulcers. Ther. Deliv..

[66] Capanema N.S.V., Mansur A.A.P., Carvalho S.M., Mansur L.L., Ramos C.P., Lage A.P., Mansur H.S. (2018). Physicochemical properties and antimicrobial activity of biocompatible carboxymethylcellulose-silver nanoparticle hybrids for wound dressing and epidermal repair. J. Appl. Polym. Sci..

[67] Yao C.H., Lee C.Y., Huang C.H., Chen Y.S., Chen K.Y. (2017). Novel bilayer wound dressing based on electrospun gelatin/keratin nanofibrous mats for skin wound repair. Mater. Sci. Eng. C-Mater. Biol. Appl..

[68] Cerchiara T., Abruzzo A., Ñahui Palomino R.A., De Rose B.V.R., Chidichimo G., Ceseracciu L., Athanassiou A., Saladini B., Dalena F., Bigucci F. (2017). Spanish Broom (*Spartium junceum* L.) fibers impregnated with vancomycin-loaded chitosan nanoparticles as new antibacterial wound dressing: Preparation, characterization and antibacterial activity. Eur. J. Pharm. Sci..

[69] Swenty C.F. (2016). Principles to Guide Your Dressing Choice. J. Nurse Pract..

[70] Asfaw T., Jackson J.C., Lu Z., Zhai X., Shums S., Hirt T., Hu X., René C.R. (2016). In-Situ Forming Hydrogel Wound Dressings Containing Antimicrobial Agents. U.S. Patent.

[71] Ousey K., Cutting K., Rogers A.A., Rippon M. (2016). The importance of hydration in wound healing: Reinvigorating the clinical perspective. J. Wound Care.

[72] Dhivya S., Padma V.V., Santhini E. (2015). Wound dressings—A review. Biomedicine.

[73] Qiu Y., Qiu L., Cui J., Wei Q. (2016). Bacterial cellulose and bacterial cellulose-vaccarin membranes for wound healing. Mater. Sci. Eng. C.

[74] Fan L., Yang H., Yang J., Peng M., Hu J. (2016). Preparation and characterization of chitosan/gelatin/PVA hydrogel for wound dressings. Carbohydr. Polym..

[75] Dabiri G., Damstetter E., Phillips T. (2016). Choosing a wound dressing based on common wound characteristics. Adv. Wound Care.

[76] Ramos-e-Silva M., de Castro M.C.R. (2002). New dressings, including tissue-engineered living skin. Clin. Dermatol..

[77] Flores C., Lopez M., Tabary N., Neut C., Chai F., Betbeder D., Herkt C., Cazaux F., Gaucher V., Martel B. (2017). Preparation and characterization of novel chitosan and β-cyclodextrin polymer sponges for wound dressing applications. Carbohydr. Polym..

[78] Pott F.S., Meier M.J., Stocco J.G.D., Crozeta K., Ribas J.D. (2014). The effectiveness of hydrocolloid dressings versus other dressings in the healing of pressure ulcers in adults and older adults: A systematic review and meta-analysis. Rev. Lat.-Am. Enferm..

[79] Das S., Baker A. (2016). Biomaterials and Nanotherapeutics for Enhancing Skin Wound Healing. Front. Bioeng. Biotechnol..

[80] Woundcarehandbook. http://www.woundcarehandbook.com.

[81] Sweeney I.R., Miraftab M., Collyer G. (2012). A critical review of modern and emerging absorbent dressings used to treat exuding wounds. Int. Wound J..

[82] Chattopadhyay S., Raines R.T., Glick G.D. (2014). Review collagen-based biomaterials for wound healing. Biopolymers.

[83] Calo E., Ballamy L., Khutoryanskiy V.V., Singh T.R.R., Leverty G., Donelly R. (2018). Hydrogels in Wound Management. Hydrogels: Design, Synthesis and Application in Drug Delivery and Regenerative Medicine.

[84] Vermeulen H., Ubbink D.T., Goossens A., de Vos R., Legemate D.A. (2005). Systematic review of dressings and topical agents for surgical wounds healing by secondary intention. Br. J. Surg..

[85] Terry Swanson N.P.W.M., International Wound Infection Institute (IWII) (2016). Wound Infection in Clinical Practice.

[86] Liu X., Nielsen L.H., Kłodzińska S.N., Nielsen H.M., Quc H., Christensen L.P., Rantanen J., Yangad M. (2018). Ciprofloxacin-loaded sodium alginate/poly(lactic-*co*-glycolic acid) electrospun fibrous mats for wound healing. Eur. J. Pharm. Biopharm..

[87] Contardi M., Heredia-Guerrero J.A., Perotto G., Valentini P., Pompa P.P., Spanò R., Goldonic L., Bertorelli R., Athanassiou A., Bayera I.S. (2017). Transparent ciprofloxacin-povidone antibiotic films and nanofiber mats as potential skin and wound care dressings. Eur. J. Pharm. Sci..

[88] Li H., Williams G.R., Wang J.W.H., Sun X., Zhu L.M. (2017). Poly(*N*-isopropylacrylamide)/poly(l-lactic acid-*co*-ɛ-caprolactone) fibers loaded with ciprofloxacin as wound dressing materials. Mater. Sci. Eng. C Mater. Biol. Appl..

[89] Pamfil D., Vasile C., Tarţău L., Vereştiuc L., Poiată A. (2017). pH-Responsive 2-hydroxyethyl methacrylate/citraconic anhydride–modified collagen hydrogels as ciprofloxacin carriers for wound dressings. J. Bioact. Compat. Polym..

[90] Khampieng T., Wnek G.E., Supaphol P. (2014). Electrospun DOXY-h loaded-poly(acrylic acid) nanofiber mats: In vitro drug release and antibacterial properties investigation. J. Biomater. Sci.-Polym. Ed..

[91] Michalska-Sionkowska M., Kaczmarek B., Walczak M., Sionkowska A. (2018). Antimicrobial activity of new materials based on the blends of collagen/chitosan/hyaluronic acid with gentamicin sulfate addition. Mater. Sci. Eng. C Mater. Biol. Appl..

[92] Ahire J.J., Robertson D.D., van Reenen A.J., Dicks L.M.T. (2017). Polyethylene oxide (PEO)-hyaluronic acid (HA) nanofibers with kanamycin inhibits the growth of Listeria monocytogenes. Biomed. Pharmacother..

[93] Etebu E., Arikekpar I. (2016). Antibiotics: Classification and mechanisms of action with emphasis on molecular perspectives. Int. J. Appl. Microbiol. Biotechnol. Res..

[94] Kohanski M.A., Dwyer D.J., Collins J.J. (2010). How antibiotics kill bacteria: From targets to networks. Nat. Rev. Microbiol..

[95] Rai M., Kon K., Gade A., Ingle A., Nagaonkar D., Paralikar P., da Silva S.S. (2016). Chapter 6—Antibiotic Resistance: Can Nanoparticles Tackle the Problem?. Antibiotic Resistance. Mechanisms and New Antimicrobial Approaches.

[96] Friedman N.D., Temkin E., Carmeli Y. (2016). The negative impact of antibiotic resistance. Clin. Microbiol. Infect..

[97] Pîrvănescu H., Bălăşoiu M., Ciurea M.E., Bălăşoiu A.T., Mănescu R. (2014). Wound infections with multi-drug resistant bacteria. Chirurgia.

[98] Chávez-González M.L., Rodríguez-Herrera R., Aguilar C.N. (2016). Chapter 11—Essential Oils: A Natural Alternative to Combat Antibiotics Resistance. Antibiotic Resistance. Mechanisms and New Antimicrobial Approaches.

[99] Shrestha G., Raphael J., Leavitt S.D., St Clair L.L. (2014). In vitro evaluation of the antibacterial activity of extracts from 34 species of North American lichens. Pharm. Biol..

[100] Segev-Zarko L., Saar-Dover R., Brumfeld V., Mangoni M.L., Shai Y. (2015). Mechanisms of biofilm inhibition and degradation by antimicrobial peptides. Biochem. J..

[101] Seow Y.X., Yeo C.R., Chung H.L., Yuk H.-G. (2014). Plant essential oils as active antimicrobialagents. Crit. Rev. Food Sci. Nutr..

[102] Agyare C., Duah Y., Oppong E., Hensel A., Oteng S., Appiah T. (2016). Review: African medicinal plants with wound healing properties. J. Ethnopharmacol..

[103] Semeniuc C.A., Popa C.R., Rotar A.M. (2017). Antibacterial activity and interactions of plant essential oil combinations againts Gram-positive and Gram-negative bacteria. J. Food Drug Anal..

[104] Kavoosi G., Dadfar S.M.M., Purfard A.M., Mehrabi R. (2013). Antioxidant and Antibacterial Properties of Gelatin Films Incorporated with Carvacrol. J. Food Saf..

[105] Altiok D., Altiok E., Tihminlioglu F. (2010). Physical, antibacterial and antioxidant properties of chitosan films incorporated with thyme oil for potential wound healing applications. J. Mater. Sci. Mater. Med..

[106] Walsh S.E., Maillard J.-Y., Russell A.D., Catrenich C.E., Charbonneau D.L., Bartolo R.G. (2003). Development of bacterial resistance to several biocides and effects on antibiotic susceptibility. J. Hosp. Infect..

[107] Sienkiewicz M., Głowacka A., Kowalczyk E., Wiktorowska-Owczarek A., Jóźwiak-Bębenista M., Łysakowska M. (2014). The Biological Activities of Cinnamon, Geranium and Lavender Essential Oils. Molecules.

[108] Zenati F., Benbelaid F., Khadir A., Bellahsene C., Bendahou M. (2014). Antimicrobial effects of three essential oils on multidrug resistant bacteria responsible for urinary infections. J. Appl. Pharm. Sci..

[109] Liakos I., Rizzello L., Scurr D.J., Pompa P.P., Bayer I.S., Athanassiou A. (2014). All-natural composite wound dressing films of essential oils encapsulated in sodium alginate with antimicrobial properties. Int. J. Pharm..

[110] Liakos I., Rizzello L., Hajiali H., Brunetti V., Carzino R., Pompa P., Athanassiou A., Mele E. (2015). Fibrous wound dressings encapsulating essential oils as natural antimicrobial agents. J. Mater. Chem. B.

[111] Rosa J.M., Bicudo Bonato L., Bragine Mancuso C., Martinelli L., Okura M.H., Malpass G.R.P., Granato A.C. (2018). Antimicrobial wound dressing films containing essential oils and oleoresins of pepper encapsulated in sodium alginate films. Cienc. Rural.

[112] Nogueira M.N.M., Aquino S.G., Rossa Junior C., Spolidorio D.M.P. (2014). Terpinen-4-ol and alpha-terpineol (tea tree oil components) inhibit the production of IL-1b, IL-6 and IL-10 on human macrophages. Inflamm. Res..

[113] Bai M.-Y., Chou T.-C., Tsai J.-C., Yu W.-C. (2014). The effect of active ingredient-containing chitosan/polycaprolactone nonwoven mat on wound healing: In vitro and in vivo studies. J. Biomed. Mater. Res. Part A.

[114] Ge Y., Ge M. (2015). Sustained broad-spectrum antimicrobial and haemostatic chitosan-based film with immerged tea tree oil droplets. Fibers Polym..

[115] Edmondson M., Newall N., Carville K., Smith J., Riley T.V., Carson C.F. (2011). Uncontrolled, open-label, pilot study of tea tree (*Melaleuca alternifolia*) oil solution in the decolonisation of methicillin-resistant *Staphylococcus aureus* positive wounds and its influence on wound healing. Int. Wound J..

[116] Lee R., Leung P., Wong T. (2014). A randomized controlled trial of topical tea tree preparation for MRSA colonized wounds. Int. J. Nurs. Sci..

[117] Cuttle L., Kempf M., Kravchuk O., George N., Liu P.Y., Chang H.E., Mill J., Wang X.Q., Kimble R.M. (2008). The efficacy of Aloe vera, tea tree oil and saliva as first aid treatment for partial thickness burn injuries. Burns.

[118] Saddiqe Z., Naeem I., Maimoona A. (2010). A review of the antibacterial activity of *Hypericum perforatum* L.. J. Ethnopharmacol..

[119] Güneş S., Tıhmınlıoğlu F. (2017). Hypericum perforatum incorporated chitosan films as potential bioactive wound dressing material. Int. J. Biol. Macromol..

[120] Evandri M.G., Battinelli L., Daniele C., Mastrangelo S., Bolle P., Mazzanti G. (2005). The antimutagenic activity of *Lavandula angustifolia* (lavender) essential oil in the bacterial reverse mutation assay. Food Chem. Toxicol..

[121] Cavanagh H.M.A., Wilkinson J.M. (2002). Biological activities of lavender essentials oil. Phytother. Res..

[122] Imane M.M., Houda F., Amal A.H.S., Kaotar N., Mohammed T., Imane R., Farid H. (2017). Phytochemical Composition and Antibacterial Activity of Moroccan *Lavandula angustifolia* Mill. J. Essent. Oil Bear. Plants.

[123] Mori H., Kawanami H., Kawahata H., Aoki M. (2016). Wound healing potential of lavender oil by acceleration of granulation and wound contraction through induction of TGF-β in a rat model. BMC Complement. Altern. Med..

[124] Sarikurkcu C., Zengin G., Oskay M., Uysal S., Ceylan R., Aktumsek A. (2015). Composition, antioxidant, antimicrobial and enzyme inhibition activities of two *Origanum vulgare* subspecies (subsp. *vulgare* and subsp. *hirtum*) essential oils. Ind. Crops Prod..

[125] Nostro A., Blanco A.R., Cannatelli M.A., Enea V., Flamini G., Morelli I., Sudano Roccaro A., Alonzo V. (2004). Susceptibility of methicillin-resistant staphylococci to oregano essential oil, carvacrol and thymol. FEMS Microbiol. Lett..

[126] Liakos I.L., Holban A.M., Carzino R., Lauciello S., Grumezescu A.M. (2017). Electrospun Fiber Pads of Cellulose Acetate and Essential Oils with Antimicrobial Activity. Nanomaterials.

[127] Bogdanov S. The Bee Products: The Wonders of the Bee Hexagon, Bee Product Science. www.bee-hexagon.net.

[128] Kwakman P.H., te Velde A.A., de Boer L., Speijer D., Vandenbroucke-Grauls C.M., Zaat S.A. (2010). How honey kills bacteria. FASEB J..

[129] Molan P.C. (2006). The evidence supporting the use of honey as a wound dressing. Int. J. Lower Extrem. Wounds.

[130] Israili Z.H. (2014). Antimicrobial properties of honey. Am. J. Ther..

[131] Boateng J., Diunase K.N. (2015). Comparing the antibacterial and functional properties of cameroonian and manuka honeys for potential wound healing—Have we come full cycle in dealing with antibiotic resistance?. Molecules.

[132] Simon A., Traynor K., Santos K., Blaser G., Bode U., Molan P. (2009). Medical honey for wound care—Still the ‘latest resort’?. Evid.-Based Complement. Altern. Med..

[133] Kuś P.M., Szweda P., Jerković I., Tuberoso C.I. (2016). Activity of Polish unifloral honeys against pathogenic bacteria and its correlation with colour, phenolic content, antioxidant capacity and other parameters. Lett. Appl. Microbiol..

[134] Sherlock O., Dolan A., Athman R., Power A., Gethin G., Cowman S., Humphreys H. (2010). Comparison of the antimicrobial activity of Ulmo honey from Chile and Manuka honey against methicillin-resistant *Staphylococcus aureus*, *Escherichia coli* and *Pseudomonas aeruginosa*. BMC Complement. Altern. Med..

[135] Ng W.J., Lim M.S. (2015). Anti-staphylococcal activity of melaleuca honey. Southeast Asian J. Trop. Med. Public Health.

[136] Jantakee K., Tragoolpua Y. (2015). Activities of different types of Thai honey on pathogenic bacteria causing skin diseases, tyrosinase enzyme and generating free radicals. Biol. Res..

[137] Packer J.M., Irish J., Herbert B.R., Hill C., Padula M., Blair S.E., Carter D.A., Harry E.J. (2012). Specific non-peroxide antibacterial effect of manuka honey on the *Staphylococcus aureus* proteome. Int. J. Antimicrob. Agents.

[138] Cooper R. (2014). Honey as an effective antimicrobial treatment for chronic wounds: Is there a place for it in modern medicine?. Chronic Wound Care Manag. Res..

[139] Bulman S.E., Tronci G., Goswami P., Carr C., Russell S.J. (2017). Antibacterial properties of nonwoven wound dressings coated with Manuka honey or methylglyoxal. Materials.

[140] Lu J., Turnbull L., Burke C.M., Liu M., Carter D.A., Schlothauer R.C., Whitchurch C.B., Harry E.J. (2014). Manuka-type honeys can eradicate biofilms produced by *Staphylococcus aureus* strains with different biofilm-forming abilities. PeerJ.

[141] Minden-Birkenmaier B.A., Neuhalfen R.M., Janowiak B.E., Sell S.A. (2015). Preliminary Investigation and Characterization of Electrospun Polycaprolactone and Manuka Honey Scaffolds for Dermal Repair. J. Eng. Fiber Fabr..

[142] Yang X., Fan L., Ma L., Wang Y., Lin S., Yu F., Pan X., Luo G., Zhang D., Wang H. (2017). Green electrospun Manuka honey/silk fibroin fibrous matrices as potential wound dressing. Mater. Des..

[143] Tavakoli J., Tang Y. (2017). Honey/PVA hybrid wound dressings with controlled release of antibiotics: Structural, physico-mechanical and in-vitro biomedical studies. Mater. Sci. Eng. C Mater. Biol. Appl..

[144] Saikaly S.K., Khachemoune A. (2017). Honey and Wound Healing: An Update. Am. J. Clin. Dermatol..

[145] Rai M., Yadav A., Gade A. (2009). Silver nanoparticles as a new generation of antimicrobials. Biotechnol. Adv..

[146] Kumar M., Curtis A., Hoskins C. (2018). Application of Nanoparticle Technologies in the Combat against Anti-Microbial Resistance. Pharmaceutics.

[147] Kandi V., Kandi S. (2015). Antimicrobial properties of nanomolecules: Potential candidates as antibiotics in the era of multi-drug resistance. Epidemiol. Health.

[148] Simões D., Miguel S.P., Ribeiro M.P., Coutinho P., Mendonça A.G., Correia I.J. (2018). Recent advances on antimicrobial wound dressing: A review. Eur. J. Pharm. Biopharm..

[149] Wang L., Hu C., Shao L. (2017). The antimicrobial activity of nanoparticles: Present situation and prospects for the future. Int. J. Nanomed..

[150] Baek Y.W., An Y.J. (2011). Microbial toxicity of metal oxide nanoparticles (CuO, NiO, ZnO, and Sb_2_O_3_) to *Escherichia coli*, *Bacillus subtilis*, and *Streptococcus aureus*. Sci. Total Environ..

[151] Ashkarran A.A., Ghavami M., Aghaverdi H., Stroeve P., Mahmoudi M. (2012). Bacterial effects and protein corona evaluations: Crucial ignored factors for prediction of bio-efficacy of various forms of silver nanoparticles. Chem. Res. Toxicol..

[152] Lu C., Brauer M.J., Botstein D. (2009). Slow growth induces heat-shock resistance in normal and respiratory-deficient yeast. Mol. Biol. Cell.

[153] Pramanik A., Laha D., Bhattacharya D., Pramanik P., Karmakar P. (2012). A novel study of antibacterial activity of copper iodide nanoparticle mediated by DNA and membrane damage. Colloids Surf. B.

[154] Rai M.K., Deshmukh S.D., Ingle A.P., Gade A.K. (2012). Silver nanoparticles, the powerful nanoweapon against multidrug-resistant bacteria. J. Appl. Microbiol..

[155] Anisha B.S., Biswas R., Chennazhi K.P., Jayakumar R. (2013). Chitosan-hyaluronic acid/nano silver composite sponges for drug resistant bacteria infected diabetic wounds. Int. J. Biol. Macromol..

[156] Nam G., Rangasamy S., Purushothaman B., Song J.M. (2015). The application of bactericidal silver nanoparticles in wound treatment. Nanomater. Nanotechnol..

[157] Verma J., Kanoujia J., Parashar P., Tripathi C.B., Saraf S.A. (2017). Wound healing applications of sericin/chitosan-capped silver nanoparticles incorporated hydrogel. Drug Deliv. Transl. Res..

[158] Kaba S.I., Egorova E.M. (2015). In vitro studies of the toxic effects of silver nanoparticles on HeLa and U937 cells. Nanotechnol. Sci. Appl..

[159] Zhou Y., Chen R., He T., Xu K., Du D., Zhao N., Cheng X., Yang J., Shi H., Lin Y. (2016). Biomedical potential of ultrafine Ag/AgCl nanoparticles coated on graphene with special reference to antimicrobial performances and burn wound healing. ACS Appl. Mater. Interfaces.

[160] Frankova J., Pivodova V., Vagnerova H., Juranova J., Ulrichova J. (2016). Effects of silver nanoparticles on primary cell cultures of fibroblasts and keratinocytes in a wound-healing model. J. Appl. Biomater. Funct. Mater..

[161] El-Naggar M.Y., Gohar Y.M., Sorour M.A., Waheeb M.G. (2016). Hydrogel dressing with a nano-formula against methicillin-resistant *Staphylococcus aureus* and *Pseudomonas aeruginosa* diabetic foot bacteria. J. Microbiol. Biotechnol..

[162] Jaiswal M., Koul V., Dinda A.K. (2016). In vitro and in vivo investigational studies of a nanocomposite-hydrogel-based dressing with a silver-coated chitosan wafer for full-thickness skin wounds. J. Appl. Polym. Sci..

[163] Nešović K., Kojić V., Rhee K.Y., Mišković-Stanković V. (2017). Electrochemical synthesis and characterization of silver doped poly(vinyl alcohol)/chitosan hydrogels. Corrosion.

[164] Hanif M., Juluri R.R., Fojan P., Popok V.N. (2016). Polymer films with size-selected silver nanoparticles as plasmon resonance-based transducers for protein sensing. Biointerface Res. Appl. Chem..

[165] Higa A.M., Mambrini G.P., Hausen M., Strixino F.T., Leite F.L. (2016). Ag-nanoparticle-based nano-immunosensor for anti-glutathione s-transferase detection. Biointerface Res. Appl. Chem..

[166] Wu J., Zheng Y., Song W., Luan J., Wen X., Wu Z., Chen X., Wang Q., Guo S. (2014). In situ synthesis of silver-nanoparticles/bacterial cellulose composites for slow-released antimicrobial wound dressing. Carbohydr. Polym..

[167] Radulescu M., Andronescu E., Dolete G., Popescu R.C., Fufă O., Chifiriuc M.C., Mogoantă L., Bălşeanu T.A., Mogoşanu G.D., Grumezescu A.M. (2016). Silver Nanocoatings for Reducing the Exogenous Microbial Colonization of Wound Dressings. Materials.

[168] Hajipour M.J., Fromm K.M., Ashkarran A.A., de Aberasturi D.J., de Larramendi I.R., Rojo T., Serpooshan V., Parak W.J., Mahmoudi M. (2012). Antibacterial properties of nanoparticles. Trends Biotechnol..

[169] Shafiee M.R.M., Kargar M. (2017). Synthesis of 3,4,5-substituted furan-2(5h)-ones using zno nanostructure as an efficient catalyst. Biointerface Res. Appl. Chem..

[170] Applerot G., Lellouche J., Perkas N., Nitzan Y., Gedanken A., Banin E. (2012). ZnO nanoparticle-coated surfaces inhibit bacterial biofilm formation and increase antibiotic susceptibility. RSC Adv..

[171] Mirzaei H., Darroudi M. (2017). Zinc oxide nanoparticles: Biological synthesis and biomedical applications. Ceram. Int..

[172] Khosravian P., Ghashang M., Ghayoor H. (2016). Zinc oxide/natural-zeolite composite nano-powders: Efficient catalyst for the amoxicillin removal from wastewater. Biointerface Res. Appl. Chem..

[173] Nair S., Sasidharan A., Rani V.V.D., Menon D., Nair S., Manzoor K., Raina S. (2009). Role of size scale of ZnO nanoparticles and microparticles on toxicity toward bacteria and osteoblast cancer cells. J. Mater. Sci. Mater. Med..

[174] Păunica-Panea G., Ficai A., Marin M.M., Marin Ș., Albu M.G., Constantin V.D., Dinu-Pîrvu C., Vuluga Z., Corobea M.C., Ghica M.V. (2016). New collagen-dextran-zinc oxide composites for wound dressing. J. Nanomater..

[175] Khorasani M.T., Joorabloo A., Moghaddam A., Shamsi H., Mansoori Moghadam Z. (2018). Incorporation of ZnO nanoparticles into heparinised polyvinyl alcohol/chitosan hydrogels for wound dressing application. Int. J. Biol. Macromol..

[176] Rădulescu M., Andronescu E., Cirja A., Holban A.M., Mogoantă L., Bălşeanu T.A., Bogdan C., Neagu T.P., Lascăr I., Florea D.A. (2016). Antimicrobial coatings based on zinc oxide and orange oil for improved bioactive wound dressings and other applications. Rom. J. Morphol. Embryol..

[177] Díez-Pascual A.M., Díez-Vicente A.L. (2015). Wound Healing Bionanocomposites Based on Castor Oil Polymeric Films Reinforced with Chitosan-Modified ZnO Nanoparticles. Biomacromolecules.

[178] Geraldo D.A., Needham P., Chandia N., Arratia-Perez R., Mora G.C., Villagra N.A. (2016). Green synthesis of polysaccharides-based gold and silver nanoparticles and their promissory biological activity. Biointerface Res. Appl. Chem..

[179] Khashayar P., Amoabediny G., Larijani B., Hosseini M., Verplancke R., Schaubroeck D., De Keersmaecker M., Adriaens A., Vanfleteren J. (2016). Characterization of gold nanoparticle layer deposited on gold electrode by various techniques for improved sensing abilities. Biointerface Res. Appl. Chem..

[180] Mikalauskaite A., Karabanovas V., Karpicz R., Rotomskis R., Jagminas A. (2016). Green synthesis of red-fluorescent gold nanoclusters: Characterization and application for breast cancer detection. Biointerface Res. Appl. Chem..

[181] Nicol J.R., Dixon D., Coulter J.A. (2015). Gold nanoparticle surface functionalization: A necessary requirement in the development of novel nanotherapeutics. Nanomedicine.

[182] Akturk O., Kismet K., Yasti A.C., Kuru S., Duymus M.E., Kaya F., Caydere M., Hucumenoglu S., Keskin D. (2016). Collagen/gold nanoparticle nanocomposites: A potential skin wound healing biomaterial. J. Biomater. Appl..

[183] Jayakumar R., Prabaharan M., Sudheesh Kumar P.T., Nair S.V., Tamura H. (2011). Biomaterials based on chitin and chitosan in wound dressing applications. Biotechnol. Adv..

[184] Regiel-Futyra A., Kus-Liśkiewicz M., Sebastian V., Irusta S., Arruebo M., Stochel G., Kyzioł A. (2015). Development of non cytotoxic chitosan-gold nanocomposites as efficient antibacterial materials. ACS Appl. Mater. Interfaces.

[185] Volkova N., Yukhta M., Pavlovich O., Goltsev A. (2016). Application of cryopreserved fibroblast culture with au nanoparticles to treat burns. Nanoscale Res. Lett..

[186] Martins A.F., Facchi S.P., Monteiro J.P., Nocchi S.R., Silva C.T.P., Nakamura C.V., Girotto E.M., Rubira A.F., Muniz E.C. (2015). Preparation and cytotoxicity of *N*,*N*,*N*-trimethyl chitosan/alginate beads containing gold nanoparticles. Int. J. Biol. Macromol..

